# Valuation of Neutrophil/Lymphocyte Ratio in Renal Cell Carcinoma Grading and Progression

**DOI:** 10.7759/cureus.2051

**Published:** 2018-01-10

**Authors:** Ersan Arda, Ilkan Yuksel, Basri Cakiroglu, Esra Akdeniz, Nusret Cilesiz

**Affiliations:** 1 Urology, Trakya University Medical Faculty; 2 Urology, Private Bir Nefes Hospital; 3 Urology, Hisar Intercontinental Hospital; 4 Biostatistics, Marmara University School of Medicine; 5 Urology, Taksim Eah

**Keywords:** neutrophil/lymphocyte ratio, renal cell carcinoma, size, prognosis

## Abstract

Introduction

We investigated the association of the neutrophil/lymphocyte ratio (NLR) with tumor size and Fuhrman grade in nonmetastatic renal cell carcinoma (RCC) cases.

Materials and methods

Data of nonmetastatic RCC (T1-4N0M0) cases, operated between 2010 and 2016, were retrospectively reviewed and 103 patients were included in the study. The patients were divided into two groups according to tumor diameter (Group 1 T < 4 cm, Group 2 T ≥ 4 cm) and into three groups according to Fuhrman grade. Twenty-eight patients with a tumor diameter of 4 cm or less in Group 1 and 75 patients with a tumor diameter greater than 4 cm in Group 2 were compared. In both grouping systems, the NLR, mean platelet volume (MPV), red cell distribution width (RDW), white blood cell (WBC), red blood cell (RBC), platelet (PLT), lymphocyte, and neutrophil values and age were compared.

Results

There were no differences in age, MPV, RDW, neutrophil, WBC, RBC, PLT counts in groups of tumor diameter (Group 1 T < 4 cm, Group 2 T ≥ 4 cm). However, the lymphocyte amount was significantly higher in cases with a tumor diameter less than 4 cm compared to the cases with a tumor diameter greater than 4 cm (p = 0.015). It was observed that the NLR had a tendency to increase in patients with tumor size greater than 4 cm compared to patients with tumor size smaller than 4 cm (p = 0.029). There were no differences in age, MPV, RDW, lymphocyte, neutrophil, WBC, RBC, PLT counts, and the NLR in different Fuhrman-graded cases.

Conclusions

There is a linear relation between the tumor size and the NLR in nonmetastatic RCC cases. Therefore, the NLR is a cheap parameter that can be used to show the tumor size, and thus it can be used to get an idea about the prognosis of the patient.

## Introduction

Renal cell carcinoma (RCC) is the most common primary renal tumor and originates from the renal cortex [1]. Along with improvements in imaging techniques, there has been a rapid increase in the incidence of this condition in the past 30 years [2]. Considering the treatment options, surgical resection is shown as the gold standard treatment for patients with clinically localized disease. However, recurrence is observed in 10-20% of patients after the surgery [3-5].

The neutrophil/lymphocyte ratio (NLR) is an easily measurable and inexpensive systemic inflammation marker. A number of malignancies have been linked to the NLR [6-7]. It has been hypothesized that the synthesis of inflammatory cytokines triggered by the tumor microenvironment alters acute phase reactants and haematological components including serum neutrophil and lymphocyte counts [8-9].

In this study, we aimed to investigate the association of the NLR with tumor size and Fuhrman grade, which are important parameters in RCC staging and prognosis in nonmetastatic RCC cases.

## Materials and methods

Study population and protocol

Patients who underwent radical and partial nephrectomy surgery between January 2010 and December 2016 were studied retrospectively. A total of 103 patients with nonmetastatic (T1-4N0M0) RCC were included in the study. The demographic information of the patients, perioperative laboratory parameters, and pathology results were recorded. The patients were divided into two groups according to tumor diameter (Group 1 T < 4 cm, Group 2 T ≥ 4 cm) and into three groups according to Fuhrman grade. In both grouping systems, the NLR, mean platelet volume (MPV), red cell distribution width (RDW), white blood cell (WBC), red blood cell (RBC), platelet (PLT), lymphocyte, and neutrophil values and age were compared.

Statistical analysis

Statistical analyses were performed using R Statistical Software (www.r-project.org) a free software environment for statistical computing and graphics.

The baseline characteristics of the groups that were continuous were presented as median, interquartile range (IQR), minimum and maximum values and those which were categorical were defined as frequencies and percentages (%). Gender was compared between tumor size groups and Fuhrman grade groups using the Fisher’s exact test. The Kolmogorov-Smirnov test was used to analyse the normality of data distribution, and as a result non-parametric tests were applied to data as the distributions were non-normal.

The baseline characteristics, age, MPV, RDW, lymphocyte, neutrophil, WBC, RBC, thrombocyte, lymphocyte, and the NLR were compared between patients with tumor size smaller than 4 cm and patients with tumor size larger than 4 cm. The Mann–Whitney U test was used for comparisons and the associated p values were given. The same group of baseline characteristics were also compared between Fuhrman grades using Kruskal-Wallis test and the associated p values were given.

Receiver operating curve (ROC) analyses were constructed to evaluate diagnostic performances and optimal cut-off values for the NLR and lymphocyte for tumor size. Youden's index, which is Maximum=Sensitivity + Specificity – 1 was used as an optimization criterion for cut-off values. The area under the receiver operating characteristic (ROC) curves was used to assess the discriminative ability of the NLR and lymphocyte for tumor size. The area underneath an ROC curve is calculated following the process outlined in Mason and Graham (2002). The standard error of area under curve (AUC) was calculated based on the Hanley and Mc Neil (1982) paper. The p-value produced for AUC is related to the Mann-Whitney U statistics. For all analyses, the p value of p < 0.05 was considered statistically significant.

## Results

Overall, 103 patients with RCC were included in the study. Frequencies and percentages for gender and Fuhrman grade are given in Table 1.

**Table 1:** Baseline characteristics of patient group.

Characteristics	Categories	n (%)
Gender	Male	61 (59)
	Female	42 (41)
Fuhrman grade	1	10 (10)
	2	48 (47)
	3	45 (43)
Tumor size	<4 cm	28 (27)
	> 4 cm	75 (73)

The Fisher’s exact test revealed that gender proportions are not statistically different in tumor size groups (p = 0.12) and gender proportions are not statistically different in Fuhrman grade groups (p = 0.381) (Table 2).

**Table 2:** Gender versus tumor size and Fuhrman grade.

Gender	TS<4 cm n (row %)	TS>4 cm n (row %)	p value	FG=1 n (row %)	FG=2 n (row %)	FG=3 n (row %)	p value
Male	13 (21)	48 (79)	0.120	4 (7)	28 (46)	29 (47)	0.381
Female	15 (36)	27 (64)		6 (14)	20 (48)	16 (38)	

The baseline characteristics of the patients with tumor size smaller than 4 cm and the patients with tumor size greater than 4 cm are summarized in Table 3.

**Table 3:** Comparison of characteristics between the patient group with tumor size 4.

Characteristics	Tumor size<4 (n=28)		Tumor size>4 (n=75)		P
	median (IQR)	min; max	median (IQR)	min; max	
Age (years)	59.5 (12.75)	34; 75	60 (15.5)	23; 86	0.203
MPV	9 (1.23)	7.2; 11.3	9 (1.7)	7; 24.6	0.683
RDW	14.95 (3.45)	12; 140	14.9 (3.45)	11.5; 154	0.659
Lymphocyte	30.35 (14.28)	8.7; 56.9	25.7 (11.58)	6.6; 64	0.015*
Neutrophil	57.65 (9.85)	28.2; 87.2	62.4 (13.2)	31.5; 108.2	0.075
WBC	6650 (2950)	589; 13000	6900 (2200)	576; 15630	0.613
RBC	4.77 (0.81)	3.86; 510	4.73 (0.80)	3.25; 627	0.386
Thrombocyte	274.5 (72.75)	109; 462	272.5 (130.5)	132; 690	0.736
NLR	1.85 (1.31)	0.496; 10.02	2.375 (1.81)	0.729; 13.61	0.029*

The comparisons were made using the Mann-Whitney U test. The lymphocyte amount for patients with tumor size smaller than 4 cm (median = 30.35; IQR = 14.28) is significantly higher than patients with tumor size larger than 4 cm (median = 25.7; IQR = 11.58). The NLR has been shown to increase in patients with tumor size greater than 4 cm compared to patients with tumor size smaller than 4 cm.

The baseline characteristics of the patients in different Fuhrman grade groups and the associated Kruskal-Wallis test p values for comparisons of groups are summarized in Table 4.

**Table 4:** Comparison of characteristics between patients with different Fuhrman grades.

Characteristics	FG=1 (n=10)		FG=2 (n=48)		FG=3 (n=45)		p
	median (IQR)	min; max	median (IQR)	min; max	median (IQR)	min; max	
Age, years	60 (12)	46; 68	60 (12.5)	23; 79	59 (17)	36; 86	0.888
MPV	8.9 (1)	7.7; 10	9.1 (1.3)	7.8; 11.6	8.8 (2.1)	7; 24.6	0.264
RDW	15.1 (2.38)	13.7; 137	14.9 (3.53)	11.9; 140	14.9 (5.2)	11.5; 154	0.669
Lymphocyte	33.7 (9.1)	7.1; 42.9	26.9 (11.3)	6.6; 64	26.6 (12.25)	7.8; 26.1	0.228
Neutrophil	56.5 (7.78)	46.7; 84.7	62.9 (13.2)	28.2; 108	61.6 (10.6)	31.5; 88.3	0.571
WBC	6100 (2500)	1133; 10900	6950 (2230)	576; 14100	6840 (2500)	579; 15630	0.794
RBC	5.09 (0.30)	4.38; 488	4.72 (0.81)	3.8; 510	4.74 (1.17)	3.25; 627	0.398
Thrombocyte	273.5 (52.8)	137; 462	265 (72.75)	132; 503	284.5 (149.5)	109; 690	0.715
NLR	1.71 (0.67)	1.09; 11.93	2.30 (1.22)	0.50; 13.61	2.30 (2.01)	0.73; 11.32	0.280

There were no differences between patients in different Fuhrman grade groups in terms of age, MPV, RDW, lymphocyte, neutrophil, WBC, RBC, thrombocyte, and the NLR.

There was a significant difference between the patient group and the control group with respect to biomarkers NLR and lymphocyte. Thus, these biomarkers were further investigated for potential cut-off points and AUC. Also, the characteristics such as sensitivity, specifity, positive predictive value, and negative predictive value for these biomarkers are given in Table 5.

**Table 5:** AUC values and cut-off points to predict tumor size.

	Cut-off point	±SE	95% CI	p-value	Sensitivity (%)	Specificity (%)	PPV (%)	NPV (%)
Lymphocyte	28	±0.063	0.533; 0.781	0.007*	58.1	71.4	84.3	39.2
NLR	2.26	±0.064	0.516; 0.766	0.015*	56.8	71.4	84	39.5

**Figure 1:**
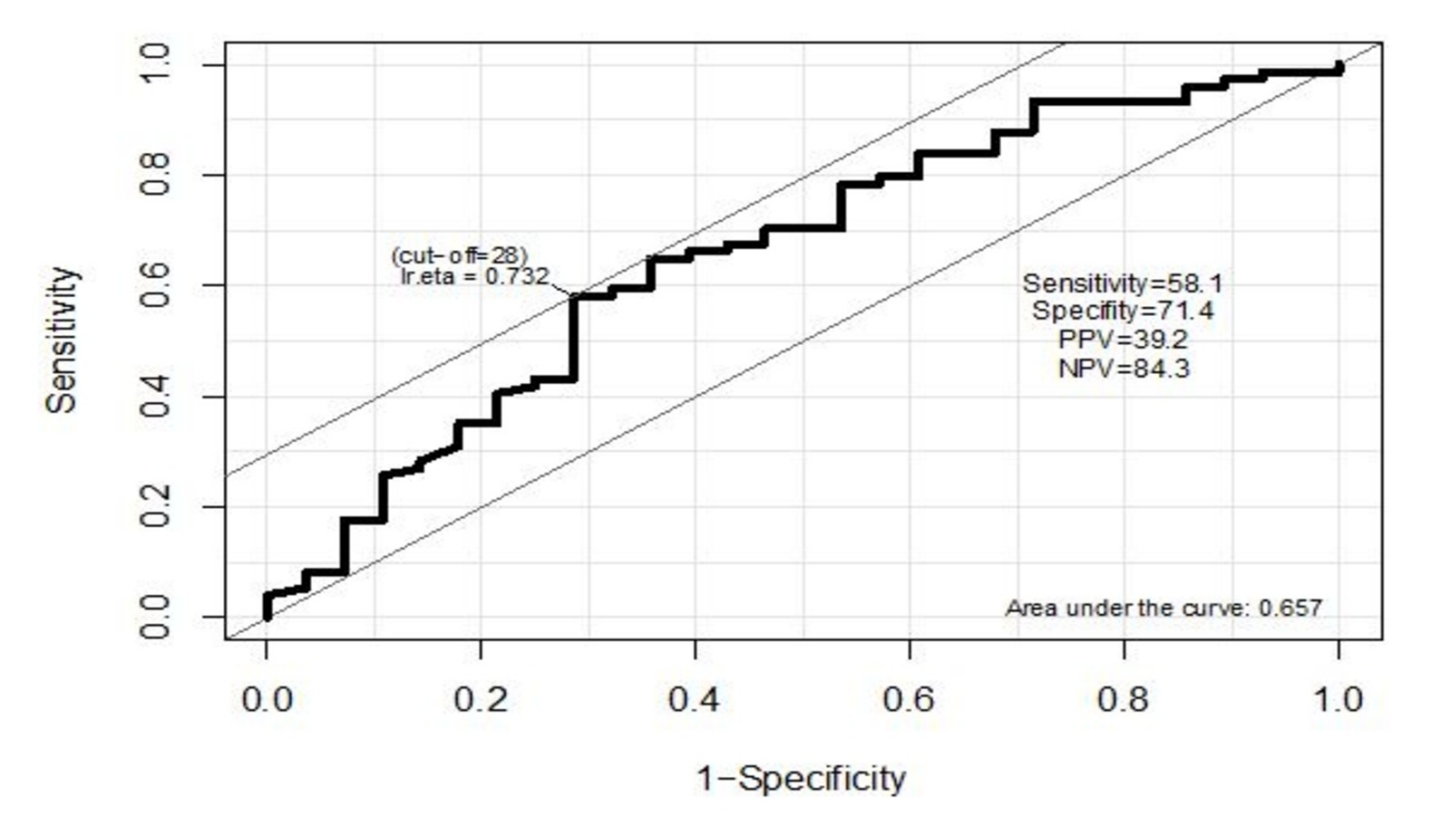
ROC curve to predict tumor size based on lymphocyte ratio. ROC - receiver operating curve

The area under the curve for the NLR is AUC = 0.641 with SE = 0.064 and 95% confidence interval (CI) from 0.516 to 0.766. The best cut-off for the NLR is 2.26. The tumor size is predicted to be larger than 4 cm if the NLR is equal to or greater than 2.26. At this cut-off point, the sensitivity is 56.8%, specificity is 71.4%, positive predictive value is 84%, and negative predictive value is 39.5% (Figure 1).

**Figure 2:**
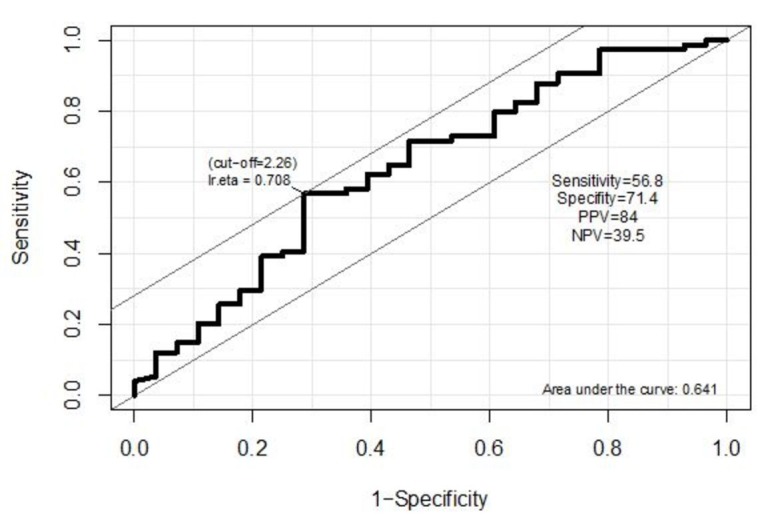
ROC curve to predict tumor size based on NLR. ROC - receiver operating curve. NLR - neutrophil/lymphocyte ratio.

The area under the curve for lymphocyte is AUC = 0.748 with SE = 0.063 and 95% CI from 0.533 to 0.781. The best cut-off for lymphocyte is 28. The tumor size is predicted to be smaller than 4 cm if lymphocyte is equal to or greater than 28. At this cut-off point, the sensitivity is 58.1%, specificity is 71.4%, positive predictive value is 84.3%, and negative predictive value is 39.2% (Figure 2).

## Discussion

Increasing evidence supports the association between inflammation and cancer development and progression [8]. Systemic inflammatory markers such as C-reactive protein (CRP), fibrinogen, ferritin, albumin, transferrin, and blood leukocyte components like neutrophils and lymphocytes are associated with prognosis of RCC, colorectal, and breast cancers [10-12].

Tumor size (TS) is an important factor that affects RCC staging and also treatment [13]. Especially, nephron-sparing surgery (NSS) is a good choice for tumors under 4 cm (T1a). In addition to this, either TS affecting RCC grade, or TS being an independent parameter in postoperative nomograms shows that it is an important parameter in terms of prognosis [14].

In our study, the NLR values of tumors larger than 4 cm were significantly higher than tumors smaller than 4 cm (p = 0.029). However, when the NLR and Fuhrman grade relation was examined, no difference was observed between the tumors smaller than 4 cm and the tumors larger than 4 cm (p = 0.280). According to these results, the NLR can be used as a cheap parameter to show the tumor size and to give an idea about the prognosis of the patient.

There are many studies investigating the relationship between the NLR and prognosis [15-19]. Viers et al. showed that the NLR ≥ 4 was significantly associated with worse five-year cancer-specific (66% vs. 85%) and overall survival (66% vs. 85%) in patients with localized RCC (p < 0.01). In contrast to our study, Viers et al. showed that the NLR had a significant association with Fuhrman grade [15].

Ohno et al. showed that 10-year recurrence-free survival rate for patients with a preoperative NLR ≥ 2.7 was significantly lower than that for those with a ratio of less than 2.7 with 64.4% to 83.7%, respectively (p = 0.0004). Ten year recurrence-free survival rate for patients with a preoperative NLR ≥ 2.7 and postoperative ratio of less than 2.7 was significantly lower than that for those with a preoperative and postoperative NLR ≥ 2.7 with 52.0% to 83.5%, respectively (p = 0.0487). As a summary to these results, the authors stated that the posttreatment NLR change is a significant prognostic factor for recurrence [16].

In another study including localized non-clear cell RCC patients, the effect of the NLR on five-year disease-free survival was evaluated. It was shown that with each 1.0 ratio increase, a risk of recurrence was increased by 15% (p = 0.0028). The authors concluded that the NLR is an independent prognostic factor for disease-free survival in localized non-clear cell renal cell carcinoma [17].

In a study evaluating metastatic RCC patients treated with everolimus, patients were stratified into two groups as NLR > 3 and NLR < 3 cm. Progression-free survival and overall survival was significantly less in patients with NLR > 3. It was demonstrated that the NLR has been shown as an independent prognostic factor also in metastatic patients [18].

A PubMed database review that included 15 studies showed that an NLR < 3 was predictive of a reduced risk of recurrence for localized RCC. Additionally, in metastatic or locally advanced RCC, an NLR < 3 predicted better overall survival and progression-free survival [19].

## Conclusions

In conclusion, the NLR is a cheap parameter that can be used to get an idea about the prognosis of patients with RCC. Nevertheless, there are currently no recommendations on the use of the NLR for RCC follow-up. Further randomized studies are required to validate the inclusion of the NLR in RCC nomograms.
